# Aberrant Expression of miR-362 Promotes Lung Cancer Metastasis through Downregulation of Sema3A

**DOI:** 10.1155/2018/1687097

**Published:** 2018-08-01

**Authors:** Dan Luo, Zheng Zhang, Zhao Zhang, Jia-Yue Li, Jian Cui, Wen-Pu Shi, Xi-Wen Dong, Lin Yuan, Peng Lin, Zhi-Nan Chen, Hui-Jie Bian, Zi-Ling Wang

**Affiliations:** ^1^College of Life Science and Bioengineering, School of Science, Beijing Jiaotong University, Beijing 100044, China; ^2^National Translational Science Center for Molecular Medicine, Department of Cell Biology, School of Basic Medicine, Air Force Medical University, Xi'an 710032, China; ^3^State Key Laboratory of Pharmaceutical Biotechnology and Collaborative Innovation Center of Chemistry for Life Sciences, School of Life Sciences, Nanjing University, Nanjing 210023, China; ^4^Institute for Special Environmental Biophysics, Key Laboratory for Space Bioscience and Biotechnology, School of Life Sciences, Northwestern Polytechnical University, Xi'an 710072, China; ^5^Clinical Laboratory, No.457 Hospital of PLA, Wuhan 430000, China

## Abstract

miR-362 is a recently discovered member of the microRNA family, and it modulates a variety of physical activities and plays an important role in the occurrence and development of many tumors. However, the biological functions of hsa-miR-362-5p in non-small-cell lung carcinoma (NSCLC) are unknown. Transwell assay and colony formation were used to determine the migration, invasion, and proliferation of NSCLC cells *in vitro*. A subcutaneous tumor model in nude mice was established to detect NSCLC tumor growth *in vivo*. The direct binding of miR-362 to the 3′UTR of Semaphorin 3A (Sema3A) was confirmed by luciferase reporter assay. In this study, we found that the level of miR-362 was higher in NSCLC tissues than in adjacent normal tissues and that the level of miR-362 expression was also elevated in five NSCLC cell lines (A549, 95-D, H1299, H292, and H460) relative to a human normal lung epithelial cell line (BEAS2B). Furthermore, miR-362 promoted NSCLC cell invasion, migration, and colony formation *in vitro* and tumor formation *in vivo*. Next, we identified the miR-362 target gene Sema3A, which is significantly correlated with metastasis. Sema3A expression was increased in normal tissues relative to NSCLC tissues. This result is consistent with the fact that miR-362 expression is negatively correlated with Sema3A expression in clinical tissue samples and indicated that miR-362 can regulate Sema3A expression in NSCLC cells and consequently affect NSCLC invasion, migration, and colony formation. Taken together, these findings on the newly identified miR-362/Sema3A axis elucidate the molecular mechanism of NSCLC invasion and migration and could lead to a potential therapeutic target in NSCLC treatment.

## 1. Introduction

Lung cancer is the most frequently diagnosed cancer worldwide and the leading cause of cancer-related death [1]. Non-small-cell lung cancer (NSCLC) accounts for approximately 85% of lung cancer cases. Its one-year survival rate is less than 10%, and only 17.8% of patients survive for 5 years or more after lung cancer diagnosis [2, 3]. After tumor resection, approximately 40% of phase I and 60% of phase II patients with NSCLC die of distant metastasis in 5 years [4, 5]. However, no early symptoms have been observed before metastasis. Effective markers can provide effective treatment plans for patients, when surgery, radiation, or chemotherapy is not applicable. The most suitable biomarkers may vary in different tumors, individuals, or treatment approaches (alone or in combination) [6].

In previous studies, the genes and proteins involved in the occurrence and development of NSCLC have been examined. However, the roles and significance of microRNAs (miRNAs) in NSCLC have not been well studied. miRNAs are small, noncoding RNAs. miRNAs can degrade mRNA or hinder its translation by forming base pairs and targeting mRNA to guide the formation of the silence complex (RISC) [7]. According to new studies, miRNAs play an important role in the occurrence and development of tumors [8–15]. Recent progress suggests that miRNAs are related to a series of human diseases, including cancer. Because miRNA genes are often located in the fragile sites of chromosomes in cancer patients [16], they have been considered as oncogene and/or tumor suppressors [17]. Recent progress suggests that miR-592 inhibits the proliferation, colony formation, and metastasis of NSCLC cells [18]. miR-133b inhibits cell growth and metastasis in NSCLC [19]. miR-301a is highly expressed in NSCLC tissues compared with paired nontumor tissues [20]. From public databases and datasets, we found that miR-362 performs an important role in a series of tumors. However, the function and mechanism of miR-362 in NSCLC are not very clear [9, 15, 21–24]. Therefore, we aimed to further clarify the function and mechanisms of miR-362 in NSCLC.

In this study, we investigated the biological functions and molecular mechanisms of miR-362 in NSCLC. miR-362 can promote NSCLC cell colony formation and metastasis. In addition, Sema3A is considered as a target gene of miR-362. miR-362/Sema3A may provide a promising therapeutic pathway for NSCLC treatment.

## 2. Materials and Methods

### 2.1. Cell Culture and Transfection

BEAS2B, A549, 95-D, H1299, H292, H460, and 293T cells were maintained in 1640 medium (Invitrogen Corporation) containing 10% fetal bovine serum (Invitrogen Corporation), penicillin, and streptomycin (100 mg/mL) at 37°C in a humidified atmosphere of 5% CO_2_. BEAS2B was a human normal lung epithelial cell line. A549, 95-D, H1299, H292, and H460 were NSCLC cell lines. Transfections [25] were performed using Lipofectamine 2000 (Invitrogen Corporation).

### 2.2. Patients and Clinical Specimens

NSCLC samples were collected from twenty-one paired patients who underwent surgical resection at Xijing Hospital before *cis-*plain and docetaxel chemotherapy. Informed consent was obtained from all patients, and the study was approved by the ethic committees of Xijing Hospital.

### 2.3. RNA Extraction and Quantitative RT-PCR Analysis

miRNA was isolated using Trizol [25] (Invitrogen Corporation) according to instruction manuals. Isolated RNA was reverse transcribed using a miScript II RT kit (Qiagen Corporation) to detect gene expression. The relative miRNA levels were determined by quantitative PCR with SYBR Green dye (Qiagen Corporation). PCR conditions were 5 min at 95°C, followed by 40 cycles of 95°C for 30 sec, 60.3°C for 30 sec, and 72°C for 30 sec (miR-362). The quantification of miR-362 and U6 (used as a loading control) was performed using specific primers: miR-362 upstream, 5′- AATCCTTGGAACCTAGGTGTGAGTAA-3′: U6 upstream, 5′- CTCGCTTCGGCAGCACA-3′: and U6 downstream, 5′- AACGCTTCACGAATTTGCGT-3′.

### 2.4. Cell Migration and Invasion Assay

Cells (5 × 10^4^) were seeded in the upper chamber of Transwell plates with 8 *μ*m pores [21]. The lower chambers of the Transwell plates were filled with 300 *μ*l of medium containing 10% FBS as a chemoattractant. The plates were incubated at 37°C for 20 h. Cell invasion assays were performed using the same method. The Transwell chambers were covered with 100 *μ*l of 1 : 3 Matrigel and serum-free medium mixture and 1 × 10^5^ A549 or 95-D cells were cultivated for 20 h. Cells that migrated or invaded the lower surface were stained with 0.2% crystal violet and quantified by counting five randomly selected microscope fields at 200x magnification.

### 2.5. Colony Formation Assay

For monolayer colony formation assay, 200 single cells of the indicated type were seeded into plates and grown for 2 weeks. Colonies were counted and photographed after methanol fixation and 0.2% crystal violet staining [23].

### 2.6. Tumor Formation in BALB/c Nude Mice

BALB/c nude mice (20 male/20 female, 4–6 weeks old and 16–20 g) were purchased from the Animal Center of the Fourth Military Medical University (FMMU). All animal experiments were carried out in accordance with the Guide for the Care and Use of Laboratory Animals of FMMU. A lung cancer xenograft model was established by suspending 5 × 10^6^ A549/A549 miR-362 knockout cells in 200 *μ*l of phosphate-buffered saline and subcutaneously inoculating the solution into the right flanks of nude mice [26–28]. When the tumor volume reached 100 mm^3^, the drug was administered. The concentration of *cis-*platin was 4 *μ*g/g, and the concentration of docetaxel was 8 *μ*g/g. Tumor size was monitored by measuring its length (L) and width (W) with calipers every 2 days, and the volumes were calculated using the formula (L × W^2^)/2. Mice were killed by cervical dislocation on day 42.

### 2.7. Cell Transfection

Mature miR-362-5p mimics (~20 nucleotide) and a negative control, which was confirmed not to interact with any mRNA sequences, were purchased from Shanghai GenePharma. Transfection of miRNA was performed using Lipofectamine 2000 (Invitrogen Corporation) according to the manufacturer's instructions. The vector containing the complete ORF of the Sema3A gene was purchased from GenScript Company.

### 2.8. Western Blotting

Sema3A protein levels were quantified by Western blotting analysis [29]. Cells were collected and washed twice with ice-cold PBS. Total proteins from cells were lysed with RIPA buffer (Thermo Fisher Scientific) for 30 min at 4°C. The suspension was then centrifuged at 16,000 g for 15 min at 4°C. The concentration of proteins was detected using a BCA assay kit (Sigma-Aldrich Corp.). Normalization was performed by blotting the same samples with an antibody against *β*-actin (Cell Signaling Technology Inc.).

### 2.9. Luciferase Assay

The 200-bp fragments of miR-362 targeting the 3′UTR (contained miR-362 seed sequence) [25] were amplified from cDNA prepared from A549 cells. The product was digested with NheI and XbaI (Takara Bio Inc.) and inserted into the pmirGLO miRNA reporter vector (Ambion, Thermo Fisher Scientific) downstream of the firefly luciferase gene. Plasmids harboring mutations (GTTCCTA) in the miR-362 target 3′UTR seed regions (CAAGGAT) were prepared using fusion PCR. 293T cells were seeded in 24-well plates and transfected with 0.8 *μ*g of pmirGLO and 5 nM miR-362/negative control. Approximately 48 h later, firefly and renilla luciferase activities were measured by a dual-luciferase assay (Promega Corporation).

### 2.10. Immunohistochemical Analysis

Formalin-fixed and paraffin-embedded tissue sections were deparaffinized in xylene, rehydrated in a graded alcohol series, and transferred to PBS. Sema3A immunostaining was carried out according to the manufacturer's instructions [30] (Biorbyt Ltd). The sections were incubated overnight at 4°C with the primary antibodies rabbit polyclonal anti-Sema3A (Biorbyt Ltd) diluted 1 : 100 in PBS.

### 2.11. Statistical Analyses

All results were confirmed in at least three independent experiments. All quantitative data are presented as the mean ± standard deviation (SD). Statistical analyses were carried out by Student *t-*tests or one-way ANOVA. *P* < 0.05 was considered significant [31].

## 3. Results

### 3.1. miR-362 Is Upregulated in NSCLC

**Figure 1:**
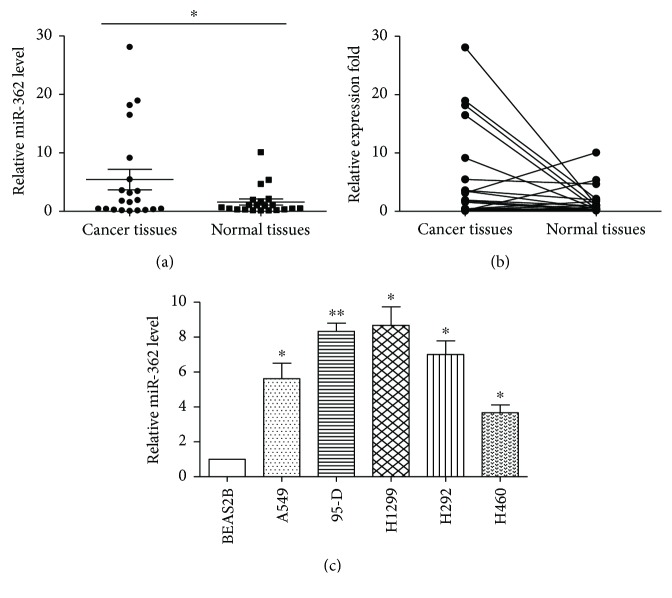
miR-362 is upregulated in NSCLC. (a) Real-time PCR analysis of the levels of miR-362 in human NSCLC tissues and adjacent noncancer tissues. (b) Comparison of miR-362 expression in 21 paired NSCLC tissues and pair-matched adjacent noncancer tissues by real-time PCR. (c) Real-time PCR analysis of the levels of miR-362 in human normal lung epithelial cells and NSCLC. The expression level of mature miR-362 is normalized by U6 small nuclear RNA. Data were from three independent experiments. ^∗^ *P* < 0.05 and ^∗∗^ *P* < 0.01.

In an attempt to explore the expression and significance of miR-362 in NSCLC, we first determined the expression of miR-362 in 21 cases of NSCLC and 21 matched adjacent normal tissue samples using quantitative reverse-transcription PCR. The results demonstrated that the expression level of miR-362 was significantly higher in NSCLC tissue than in matched normal tissue (Figures 1(a) and 1(b)). Then, we detected miR-362 expression in NSCLC cell lines (A549, 95-D, H1299, H292, and H460) and a human normal lung epithelial cell line (BEAS2B). As shown in Figure 1(c), we found that miR-362 was increased in NSCLC cell lines. H1299 and 95-D expressed higher levels of miR362 than H460 an A549.

### 3.2. miR-362 Promotes NSCLC Metastasis *In Vitro* and *In Vivo*

**Figure 2:**
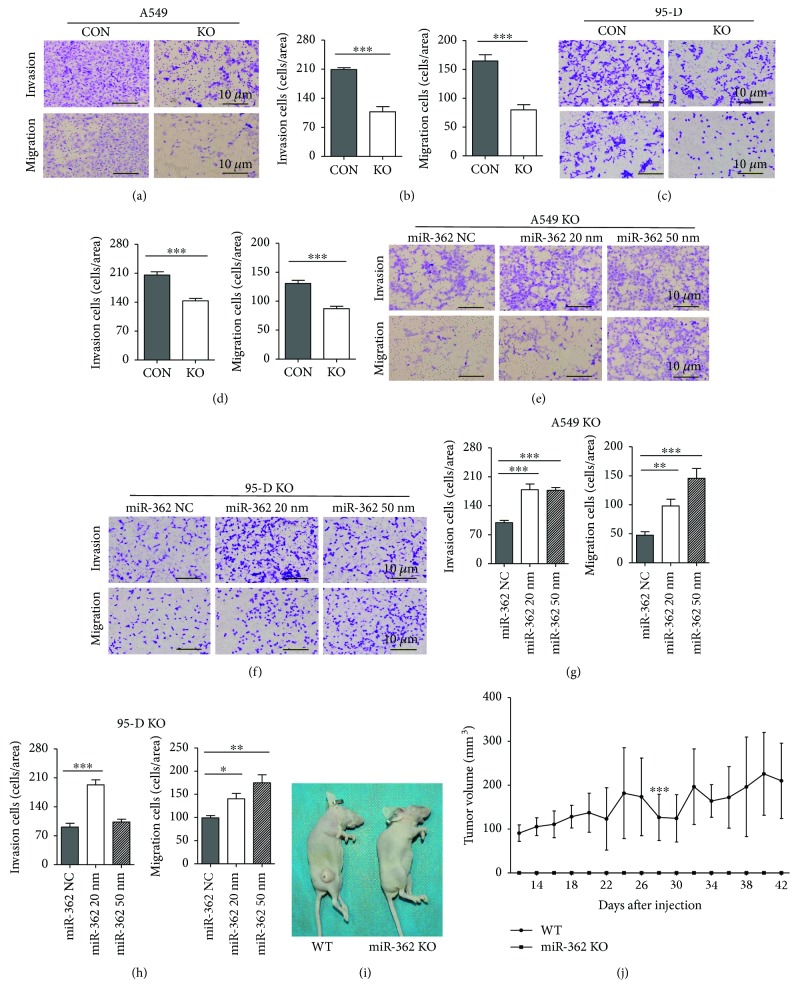
miR-362 promotes NSCLC metastasis *in vitro* and *in vivo*. (a, b) Cell invasion and migration ability decreased when miR-362 was absent in A549. (c, d) Cell invasion and migration ability decreased when miR-362 was absent in 95-D. (e, g) Transwell invasion and migration assays were employed in A549 cell lines transfected by miR-362. (f, h) Transwell invasion and migration assays were employed in 95-D cell lines transfected by miR-362. miR-362 concentrations were 20 nm and 50 nm. Cells were stained and counted using light microscopy (magnification, 200x). (i) Inhibition of miR-362 suppressed tumor formation *in vivo.* Representative images of xenograft tumors. (j) Tumor volumes were measured every two days. (a–h) Data were from three independent experiments. ^∗^ *P* < 0.05, ^∗∗^ *P* < 0.01, and ^∗∗∗^ *P* < 0.001.

We first constructed miR-362 knockout cell lines of A549 and 95-D by CRISPR/Cas9 technology to better understand the biological functions of miR-362 in the development of NSCLC [32–36]. In both A549 and 95-D cell lines, we found that knockout miR-362 significantly decreased cell invasion, migration, and colony formation ability (invasion ability, A549 in Figures 2(a) and 2(b), 95-D in Figures 2(c) and 2(d); migration ability, A549 in Figures 2(a) and 2(b), 95-D in 2(c) and 2(d); colony formation ability, A549 in Supplementary Figure 1(A, E), 95-D in Supplementary Figure 1(C, G)), whereas the cell cycle of the knockout group was not affected relative to the control group (Supplementary Figure 1(K, L)). Next, miR-362 was rescued in A549 and 95-D miR-362 knockout cell lines, which showed that miR-362 rescue significantly abolished the decreased cell invasion, migration, and colony formation in miR-362 knockout A549 and miR-362 knockout 95-D (cell invasion, A549 in Figures 2(e)and 2(g), 95-D in Figures 2(f) and 2(h); cell migration, A549 in Figures 2(e) and 2(g), 95-D in Figures 2(f) and 2(h); colony formation, A549 in Supplementary Figure 1(B, F), 95-D in Supplementary Figure 1(D, H)). The results suggested that miR-362 can promote A549 and 95-D cell invasion, migration, and colony formation.

We next tested whether blocking miR-362 activity had potential therapeutic effects in NSCLC. We established a lung cancer xenograft model to achieve this goal. After 42 days of injection, the tumor size of A549 miR-362 knockout cells was significantly smaller than that of the control groups (Figures 2(i) and 2(j)). Taken together, these observations suggest that miR-362 is a positive metastatic regulator in NSCLC.

### 3.3. miR-362 Downregulates Sema3A Expression by Directly Targeting Its 3′UTR

**Figure 3:**
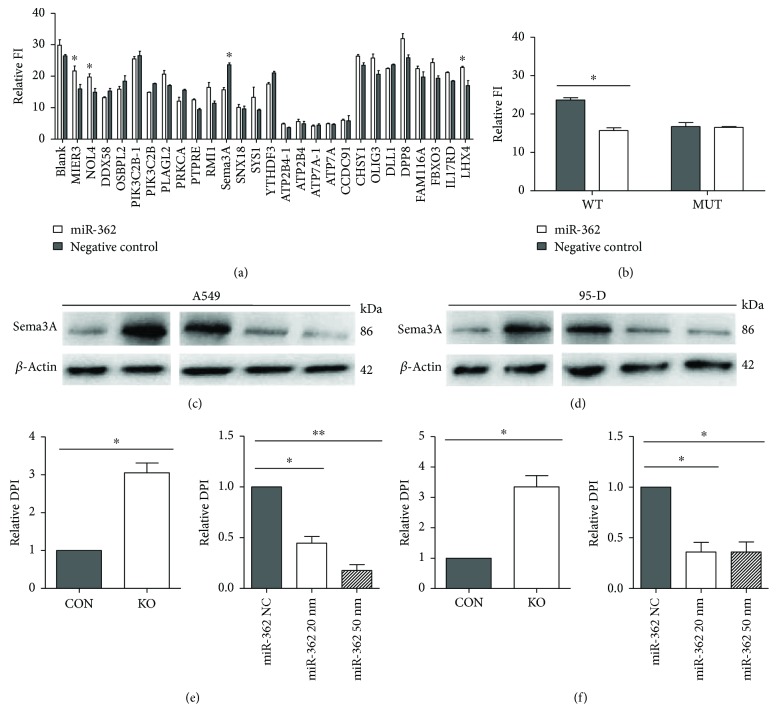
miR-362 downregulates Sema3A expression by targeting 3′UTR. (a) The relative luciferase activity of 27 candidate miR-362 targets for the 293T cell line. (b) The relative luciferase of predicted miR-362 targets Sema3A with mutated 3′UTR in 293T. (c, e) The expression of Sema3A increased when miR-362 was absent in A549 (left). Sema3A protein expression in A549 cell after transfection with NC/miR-362 (right). miR-362 concentrations were 20 nm and 50 nm. (d, f) The expression of Sema3A increased when miR-362 was absent in 95-D (left). Sema3A protein expression in 95-D cell after transfection with NC/miR-362 (right). miR-362 concentrations were 20 nm and 50 nm. Data were from three independent experiments. ^∗^ *P* < 0.05 and ^∗∗^ *P* < 0.01.

We identified 24 miR-362 candidate targets and 27 binding sites through miRanda, TargetScan, miRWalk, and miRDB software analysis (Supplementary Figure 2(A)). We separately cloned their 3′UTR into the pmirGLO vector (primers are listed in Supplementary Table 1). Dual-luciferase assay indicated that Sema3A may be a potential miR-362 regulation target (Figure 3(a)). The mutant vector, which contained the mutated binding sites of Sema3A, was constructed at the same time (Supplementary Figure 2(B)).

Then, we employed the dual-luciferase reporter assay. As expected, the relative luciferase activity of the Sema3A WT reporter was markedly reduced (66.36%) by miR-362 mimics, whereas the Sema3A MUT reporter displayed no effect relative to the control group (Figure 3(b)). This reduction was sequence specific because relative luciferase activity did not drop as sharply in UTRs that contained mutant binding sites as in those that contained wild-type binding sites. In summary, Sema3A is the target gene of miR-362. miR-362 was then knocked out in A549 and 95-D cell lines to further determine the specific target gene, and Sema3A levels were upregulated accordingly (Figures 3(c)–3(f)). After transforming miR-362 mimics into the miR-362 knockout cell lines, Sema3A levels decreased significantly (Figures 3(c)–3(f)). These data suggested that Sema3A is directly regulated by miR-362.

### 3.4. Sema3A Is Downregulated in NSCLC and Inhibits NSCLC Cell Invasion and Migration

**Figure 4:**
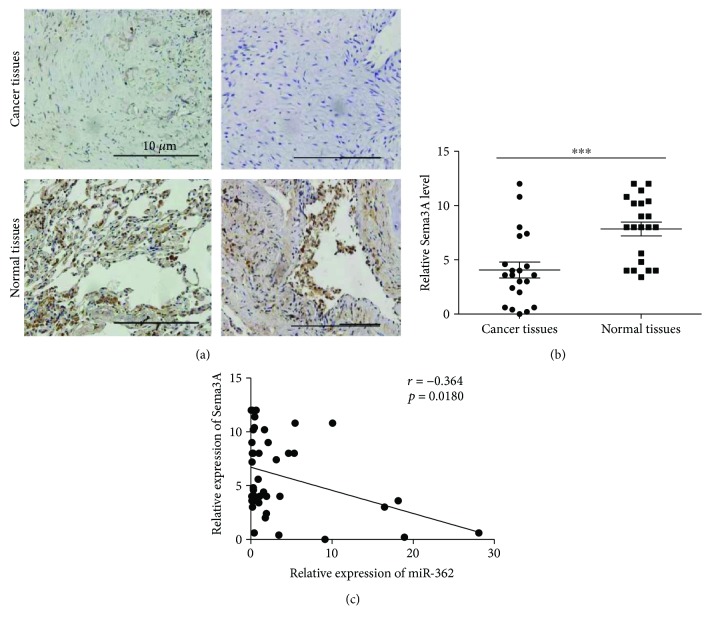
Sema3A is downregulated in NSCLC. (a, b). Sema3A levels in cancer/normal tissues in representative images 400x. (c) Correlations between the expression of miR-362 and Sema3A in NSCLC tissues. Data were from 21 cases of NSCLC. ^∗∗∗^ *P* < 0.001.

**Figure 5:**
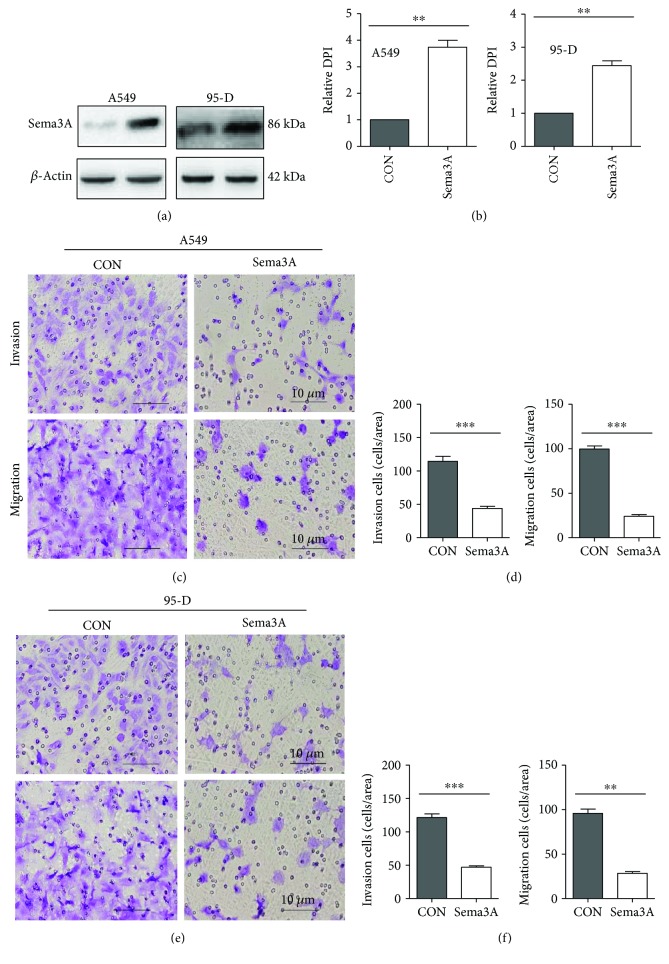
Sema3A inhibits NSCLC cell invasion and migration. (a, b) Sema3A expression in A549/95-D after transfection with the vector containing the complete ORF of the Sema3A gene or vector alone. (c, d) Cell invasion and migration ability decreased when the Sema3A vector was transfected into A549. (e, f) Cell invasion and migration ability decreased when the Sema3A vector was transfected into 95-D. Cells were stained and counted using light microscopy (magnification, 200x). Data are from three independent experiments. ^∗∗^ *P* < 0.01 and ^∗∗∗^ *P* < 0.001.

In our study, we found that the level of miR-362 was significantly higher in NSCLC tissue than in matched normal tissue (Figures 1(a) and 1(b)) and that Sema3A was directly regulated by miR-362. Previous reports indicated that decreased Sema3A expression may be associated with the development of epithelial ovarian carcinoma [37]. However, the pathological significance of Sama3A in NSCLC is still unknown. Thus, we next explored the relationship between the expression of Sema3A and miR-362 in NSCLC. First, we detected Sema3A in NSCLC and adjacent normal tissues. Our results found that the expression level of Sema3A was significantly increased in normal lung tissues (Figures 4(a) and 4(b)). This downregulation was strongly correlated with the upregulated expression of mature miR-362 in 21 cases of NSCLC tissues (Figure 4(c)). Further analysis of 21 paired NSCLC samples showed a significant upregulation of miR-362 expression and downregulation of Sema3A in cancer tissues compared with the matched noncancer tissues. These results indicated that the miR-362 level was negatively correlated with Sema3A protein expression. A549/95-D cells were transfected with a vector containing the complete ORF of the Sema3A gene or vector alone to explore the biological functions of Sema3A in NSCLC cells (Figures 5(a) and 5(b)). Remarkably, overexpression of Sema3A can strongly inhibit NSCLC cell invasion and migration (cell invasion, A549 in Figures 5(c) and 5(d), 95-D in Figures 5(e) and 5(f); cell migration, A549 in Figures 5(c) and 5(d), 95-D in Figures 5(e) and 5(f)). This phenotype was similar to the one induced by the knockout of miR-362. These data suggested the miR-362 mediated cell invasion and migration via Sema3A.

## 4. Discussion

In the present study, we demonstrated that miR-362 is commonly upregulated in NSCLC and that this upregulation can promote NSCLC cell invasion and migration both *in vitro* and *in vivo*. The target gene, Sema3A, is frequently repressed and functions as a metastasis suppressor in NSCLC; it has been confirmed to be the target gene of miR-362. Taken together, these findings indicate that miR-362 plays a fundamental role in NSCLC, especially in the process of NSCLC metastasis. This study clarified the basic functions of miR-362 and found its target gene, Sema3A; however, the underlying molecular pathway of Sema3A, the higher expression of miR-362 in cancer, and the mechanism of miR-362 upregulation are still unclear. There are potential regulatory mechanisms that explain how miR-362 modulates Sema3A. miRNA-362 is not fully complementary to Sema3A mRNA and inhibits its expression at the level of protein translation. It is also possible that miRNA-362 normally binds to the 3′UTR of Sema3A mRNA, affects the stability of Sema3A mRNA, and suppresses the translation process or directly degrades the mRNA [7]. The detailed mechanism requires further study. Also, these results are for NSCLC, but the role of miR-362 in A549 and 95-D may not be the same. Knockout miR-362 significantly increased cell apoptosis in A549, whereas cell apoptosis of the knockout group was not affected in comparison with the control group in 95-D (Supplementary Figure 1(I, J)). The detailed mechanism requires further study.

Metastasis is the main lethal factor in malignant cancer, especially in NSCLC. Hence, the identification of metastatic factors and an understanding of the molecular mechanisms related to metastasis are central issues. Recent studies have shown that miRNAs play an important role in the metastasis of NSCLC, thereby opening a new way to study the molecular mechanisms of NSCLC and developing treatment strategies against NSCLC. miR-362-5p could be used as a tumor suppressor by targeting PIK3C2B, which inhibited the proliferation and migration of neuroblastoma [21]. miR-362-3p targeted by CD82 induced tumor formation, affected epithelial-mesenchymal transition (EMT) processes, and promoted the migration and invasion of gastric cancer cells [22]. miR-362-3p targeted Tob2 affected cell cycle, proliferation, and anchorage-independent growth [23]. miR-362 promoted hepatocellular cancer proliferation and metastasis [21]. miR-362 targeted cylindromatosis (CYLD) activation of the NF-*κ*B pathway induced cell growth and apoptosis tolerance in gastric cancer. miR-362 induced cell cycle arrest in colon cancer [24]. However, there are no reports of miR-362 in NSCLC. In this study, miR-362 was shown to dramatically promote NSCLC cell metastasis. miR-362 is localized at chromosomal region Xp11.23. The upregulation of miR-362 in NSCLC tissues might be due to chromosomal acquisition of this locus. The detailed mechanism requires further exploration.

Semaphorins form a large family of at least 30 members, and they are divided into 3 types: secretory, transmembrane and GPI-anchored, and 8 subgroups. Semaphorin 3a (Sema3A) is an important protein molecule. It is found in the central nervous system and other tissues and is closely related to axon guidance, cell migration, tumor growth, immune response, and angiogenesis. It was found to involve a novel mechanism of regulating tumor suppression by Sema3A in coordination with a chain of tumor-suppressor genes, which in turn inhibits breast cancer cell migration, tumor growth, and angiogenesis [37]. Other findings have suggested that decreased Sema3A expression may be associated with the development of epithelial ovarian carcinoma, and therefore, Sema3A may be a valuable prognostic marker and a potential molecular therapy target for ovarian cancer patients [38].

## 5. Conclusions

In conclusion, we have identified that miR-362 can promote NSCLC cell metastasis and that the downregulation of Sema3A, which is a target of miR-362, can inhibit NSCLC cell metastasis. miR-362/Sema3A may provide a promising therapeutic pathway for NSCLC treatment, particularly in metastasis, and represents a candidate therapeutic target of NSCLC.

## Data Availability

All data used to support the findings of this study are included either in this article or in the supplementary information files.

## Supplementary Materials

Supplementary Materials Supplementary Figure 1: miR-362 affects other functions in NSCLC. (A, E) Cell colony formation ability decreased when miR-362 was absent in A549. (C, G) Cell colony formation ability decreased when miR-362 was absent in 95-D. (B, F) Colony formation assays were employed in A549 cell line transfected by miR-362. (D, H) Colony formation assays were employed in 95-D cell line transfected by miR-362. miR-362 concentration was 50 nm. (I, J) The cell apoptosis rate changes when miR-362 was absent in A549 and 95-D. (K, L) The cell cycle changed when miR-362 was absent in A549 and 95-D. Data are from three independent experiments. Supplementary Figure 2: prediction of miR-362 target genes. (A) The prediction of miR-362 targets by miRanda, TargetScan, miRWalk, and miRDB software. (B) Reporter vector constructs containing the Sema3A portion of the 3′UTR with the miR-362 binding site and mutated binding site. Supplemental Table 1: qRT-PCR primers of genes.

## References

[1] Sun Y. J., Zhuo Z. L., Xian H. P., Chen K. Z., Yang F., Zhao X. T. (2017). Shp2 regulates migratory behavior and response to EGFR-TKIs through ERK1/2 pathway activation in non-small cell lung cancer cells. *Oncotarget*.

[2] Wu M., Sheng Z., Jiang L., Liu Z., Bi Y., Shen Y. (2017). Overexpression of RAD51B predicts a preferable prognosis for non-small cell lung cancer patients. *Oncotarget*.

[3] Li X., Wang H., Ni Q. (2017). Effects of silencing Rab27a gene on biological characteristics and chemosensitivity of non-small cell lung cancer. *Oncotarget*.

[4] Strauss G. M. (2005). Adjuvant chemotherapy of lung cancer: methodologic issues and therapeutic advances. *Hematology/Oncology Clinics of North America*.

[5] Zhao B., Han H., Chen J. (2014). MicroRNA let-7c inhibits migration and invasion of human non-small cell lung cancer by targeting ITGB3 and MAP4K3. *Cancer Letters*.

[6] Gerger A., LaBonte M., Lenz H. J. (2011). Molecular predictors of response to antiangiogenesis therapies. *Cancer Journal*.

[7] Garzon R., Calin G. A., Croce C. M. (2009). MicroRNAs in cancer. *Annual Review of Medicine*.

[8] Jemal A., Center M. M., DeSantis C., Ward E. M. (2010). Global patterns of cancer incidence and mortality rates and trends. *Cancer Epidemiology, Biomarkers & Prevention*.

[9] Ni F., Zhao H., Cui H. (2015). MicroRNA-362-5p promotes tumor growth and metastasis by targeting CYLD in hepatocellular carcinoma. *Cancer Letters*.

[10] Jung C. J., Iyengar S., Blahnik K. R. (2011). Epigenetic modulation of miR-122 facilitates human embryonic stem cell self-renewal and hepatocellular carcinoma proliferation. *PLoS One*.

[11] Kallen A. N., Zhou X. B., Xu J. (2013). The imprinted H19 lncRNA antagonizes let-7 microRNAs. *Molecular Cell*.

[12] Nicoloso M. S., Spizzo R., Shimizu M., Rossi S., Calin G. A. (2009). MicroRNAs--the micro steering wheel of tumour metastases. *Nature Reviews Cancer*.

[13] Cornett A. L., Lutz C. S. (2014). Regulation of COX-2 expression by miR-146a in lung cancer cells. *RNA*.

[14] Feng Y., Liu J., Kang Y. (2014). miR-19a acts as an oncogenic microRNA and is up-regulated in bladder cancer. *Journal of Experimental & Clinical Cancer Research*.

[15] Xia J. T., Chen L. Z., Jian W. H. (2014). MicroRNA-362 induces cell proliferation and apoptosis resistance in gastric cancer by activation of NF-*κ*B signaling. *Journal of Translational Medicine*.

[16] Calin G. A., Dumitru C. D., Shimizu M. (2002). Frequent deletions and down-regulation of micro- RNA genes miR15 and miR16 at 13q14 in chronic lymphocytic leukemia. *Proceedings of the National Academy of Sciences of the United States of America*.

[17] O'Donnell K. A., Wentzel E. A., Zeller K. I., Dang C. V., Mendell J. T. (2005). C-Myc-regulated microRNAs modulate E2F1 expression. *Nature*.

[18] Li Z., Li B., Niu L., Ge L. (2017). miR-592 functions as a tumor suppressor in human non-small cell lung cancer by targeting SOX9. *Oncology Reports*.

[19] Zhen Y., Liu J., Huang Y., Wang Y., Li W., Wu J. (2017). miR-133b inhibits cell growth, migration, and invasion by targeting MMP9 in non-small cell lung Cancer. *Oncology Research*.

[20] Shi Y. K., Zang Q. L., Li G. X., Huang Y., Wang S. Z. (2016). Increased expression of microRNA-301a in nonsmall-cell lung cancer and Its clinical significance. *Journal of Cancer Research and Therapeutics*.

[21] Wu K., Yang L., Chen J. (2015). miR-362-5p inhibits proliferation and migration of neuroblastoma cells by targeting phosphatidylinositol 3-kinase-C2*β*. *FEBS Letters*.

[22] Zhang Q. H., Yao Y. L., Wu X. Y. (2015). Anti-miR-362-3p inhibits migration and invasion of human gastric cancer cells by its target CD82. *Digestive Diseases and Sciences*.

[23] Shen H., Li W., Tian Y. (2015). Upregulation of miR-362-3p modulates proliferation and anchorage-independent growth by directly targeting Tob2 in hepatocellular carcinoma. *Journal of Cellular Biochemistry*.

[24] Christensen L. L., Tobiasen H., Holm A. (2013). MiRNA-362-3p induces cell cycle arrest through targeting of E2F1, USF2 and PTPN1 and is associated with recurrence of colorectal cancer. *International Journal of Cancer*.

[25] Zhang X., Schulz R., Edmunds S. (2015). MicroRNA-101 suppresses tumor cell proliferation by acting as an endogenous proteasome inhibitor via targeting the proteasome assembly factor POMP. *Molecular Cell*.

[26] Shibamori M., Sato M., Uematsu N. (2015). Rebamipide does not interfere with the antitumor effect of radiotherapy or chemotherapy in human oral tumor-bearing nude mice. *Journal of Pharmacological Sciences*.

[27] Fujimoto-Ouchi K., Sekiguchi F., Yasuno H., Moriya Y., Mori K., Tanaka Y. (2007). Antitumor activity of trastuzumab in combination with chemotherapy in human gastric cancer xenograft models. *Cancer Chemotherapy and Pharmacology*.

[28] Song W., Tang Z., Zhang D. (2014). Anti-tumor efficacy of c(RGDfK)-decorated polypeptide-based micelles co-loaded with docetaxel and cisplatin. *Biomaterials*.

[29] Zhang Y., Liu D., Chen X. (2010). Secreted monocytic miR-150 enhances targeted endothelial cell migration. *Molecular Cell*.

[30] Nozawa H., Tadakuma T., Ono T. (2006). Small interfering RNA targeting epidermal growth factor receptor enhances chemosensitivity to cisplatin, 5-fluorouracil and docetaxel in head and neck squamous cell carcinoma. *Cancer Science*.

[31] Zhou W., Fong M. Y., Min Y. (2014). Cancer-secreted miR-105 destroys vascular endothelial barriers to promote metastasis. *Cancer Cell*.

[32] Bassett A. R., Liu J. L. (2014). CRISPR/Cas9 and genome editing in Drosophila. *Journal of Genetics and Genomics*.

[33] Wang H., Yang H., Shivalila C. S. (2013). One-step generation of mice carrying mutations in multiple genes by CRISPR/Cas-mediated genome engineering. *Cell*.

[34] Pelletier S., Gingras S., Green D. R. (2015). Mouse genome engineering via CRISPR-Cas9 for study of immune function. *Immunity*.

[35] Ran F. A., Hsu P. D., Wright J., Agarwala V., Scott D. A., Zhang F. (2013). Genome engineering using the CRISPR-Cas9 system. *Nature Protocols*.

[36] Shen B., Zhang W., Zhang J. (2014). Efficient genome modification by CRISPR-Cas9 nickase with minimal off-target effects. *Nature Methods*.

[37] Mishra R., Thorat D., Soundararajan G. (2015). Semaphorin 3A upregulates FOXO 3a-dependent MelCAM expression leading to attenuation of breast tumor growth and angiogenesis. *Oncogene*.

[38] Jiang H., Qi L., Wang F., Sun Z., Huang Z., Xi Q. (2015). Decreased semaphorin 3A expression is associated with a poor prognosis in patients with epithelial ovarian carcinoma. *International Journal of Molecular Medicine*.

