# Supplementary material for: Aberrant Expression of miR-362 Promotes Lung Cancer Metastasis through Downregulation of Sema3A
Source: J Immunol Res. 2018 Aug 1;2018:1687097. doi: 10.1155/2018/1687097 (PMC6093061; doi:10.1155/2018/1687097)
Supplement: Supplementary Materials — Supplementary Figure 1: miR-362 affects other functions in NSCLC. (A, E) Cell colony formation ability decreased when miR-362 was absent in A549. (C, G) Cell colony formation ability decreased when miR-362 was absent in 95-D. (B, F) Colony formation assays were employed in A549 cell line transfected by miR-362. (D, H) Colony formation assays were employed in 95-D cell line transfected by miR-362. miR-362 concentration was 50 nm. (I, J) The cell apoptosis rate changes when miR-362 was absent in A549 and 95-D. (K, L) The cell cycle changed when miR-362 was absent in A549 and 95-D. Data are from three independent experiments. Supplementary Figure 2: prediction of miR-362 target genes. (A) The prediction of miR-362 targets by miRanda, TargetScan, miRWalk, and miRDB software. (B) Reporter vector constructs containing the Sema3A portion of the 3′UTR with the miR-362 binding site and mutated binding site. Supplemental Table 1: qRT-PCR primers of genes. [file 1687097.f1.zip › Supplementary Materials_JIR_2352099.pdf]

Supplementary Fig. 1 **miR-362 affects other functions in NSCLC.** AE. Cell colony formation ability decreased when miR-362 was absent in A549. CG. Cell colony formation ability decreased when miR-362 was absent in 95-D. BF. Colony formation assays were employed in A549 cell line transfected by miR-362. DH. Colony formation assays were employed in 95-D cell line transfected by miR-362. miR-362 concentration was 50 nm. IJ. The cell apoptosis rate changes when miR-362 was absent in A549 and 95-D. KL. The cell cycle changed when miR-362 was absent in A549 and 95-D. Data are from three independent experiments.

Supplementary Fig. 2 **Prediction of miR-362 target genes.** A. The prediction of miR-362 targets by miRanda, TargetScan, miRwalk and miRDB software. B. Reporter vector constructs containing the Sema3A portion of the 3'UTR with the miR-362 binding site and mutated binding site.

Supplementary Figure 1

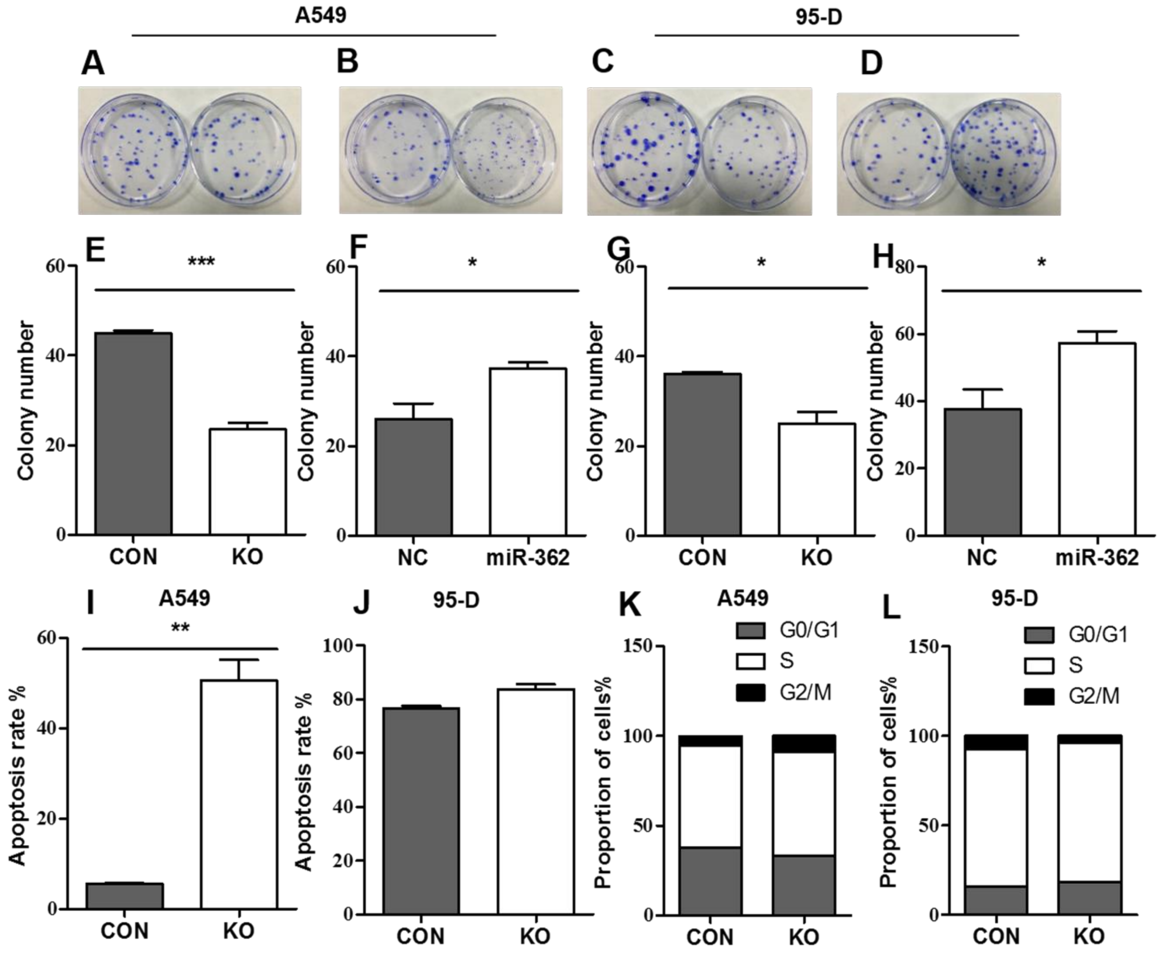

Supplementary Figure 2

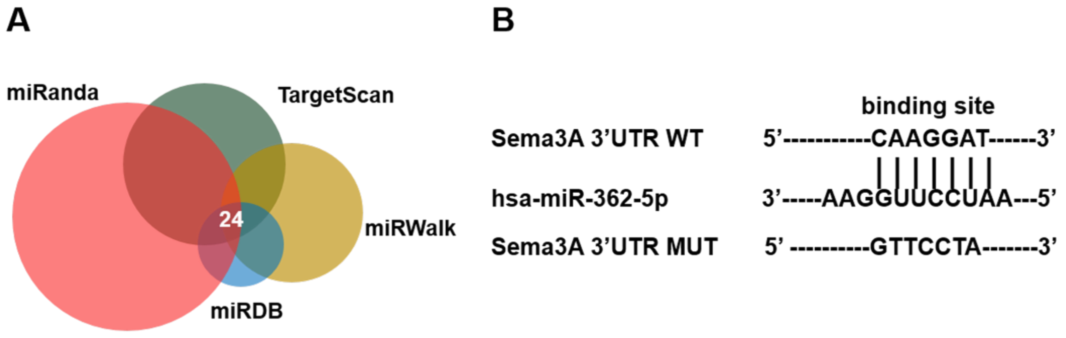

Supplemental Table1 qRT-PCR primers of genes

Primer name	Primer sequence(5'-3')
ATP2B4-F1	CTCGAGCTTTTCATTGTCACGTCAGCT
ATP2B4-R1	GTCGACATGACAAGTGACTACCAAGGAG
ATP2B4-F2	CTCGAGGGTCTATAAGGATACTCTGATT
ATP2B4-R2	GTCGACGAACCAGCCATTGTTTTATC
ATP7A-F1	CTCGAGGATATTTGAATATTTGAAGTTCTCT
ATP7A-R1	GTCGACCTACTCTAGCTGTGTTTTAATCTTT
ATP7A-F2	CTCGAGCTCAACCTTACGTTTTTAGTTAAAG
ATP7A-R2	GTCGACACACTATAATTATAAAGTTGAAGGC
CCDC91 -F	CTCGAGTGGATAAATCATATTACACCTTCA
CCDC91 -R	GTCGACGCTGCTACTACTAATAATAATAAAAT
CHSY1 -F	CTCGAGAGCTTTGCTGGAAAAGACGTT
CHSY1 -R	GTCGACCTACATAATGTTCAGCAAGAAGA
DDX58-F	CTCGAGCAATCTGGTGAGCAGTGGT
DDX58-R	GTCGACGTAAGAATACTACTACTAGTAACTATTATC
DLL1-F	CTCGAGTGAGATGGCAAGACTCCCGTT
DLL1-R	GTCGACCGGAAGGCAGTGCCGCAG
DPP8 -F	CTCGAGATAGCTTATGCTTCATTGCTT
DPP8 -R	GTCGACTATCATCAAAGAAAAATGAGAAT
FAM116A-F	CTCGAGCATCTTATTATTTTTCAAAAAGGT
FAM116A-R	GTCGACAAATGAAGACACCATTATCATG
FBXO3-F	CTCGAGTACCTTTGTTTTAAAATATTTTATACTT
FBXO3-R	GTCGACTATTAGGAATCACTAAATGGATTAG
IL17RD-F	CTCGAGAGTCTAAGCATTGCCACTTTA
IL17RD-R	GTCGACAGAGCTCCATATCATTTTAGAAA
LHX4-F	CTCGAGACCCTACCTGCCCCCCTGG
LHX4-R	GTCGACTGTGTTCTGAGATGAGTCTGCTCTC
MIER3-F	CTCGAGACCGATCCCGATCCCTGCT
MIER3-R	GTCGACGCACACAGTTCCTTGTGCTGG
NOL4-F	CTCGAGTACTTTAGTATGTCCAGCCTATTGC
NOL4-R	GTCGACTTTTTGCCAGGTACTTCAGCTT
OLIG3-F	CTCGAGGACGCAGTGTAGGTCAAAAGAAT
OLIG3-R	GTCGACGAAGCGCGTGTGCGTGAGT
OSBPL2-F	CTCGAGCACTGTGAGCCTCGTCACAGCA
OSBPL2-R	GTCGACGGCGCCGTGAAGAATTTCCCT
PIK3C2B-F1	CTCGAGGACTTTGAATTTGATATGAATCTTT
PIK3C2B-R1	GTCGACTTAAACTTCCATGCTGCCCT
PIK3C2B-F2	CTCGAGTGCTGACTCATTCCATAACTGGA
PIK3C2B-R2	GTCGACTACAGGCACAAAGCCAGTAACACA
PLAGL2-F	CTCGAGGTCCTCTCTCCACCTGCCCTCTTAT
PLAGL2-R	GTCGACAGGTTCAGAGTTCCAGGAGAGGC

continued

Primer name	Primer sequence(5’-3’)
PRKCA-F	CTCGAGAACTGTCTTGATACTTTTCATTCTT
PRKCA-R	GTCGACGTGGGTGTTGGCGAACAACA
PTPRE-F	CTCGAGTATGTTATTACAGTTTCAACTATCATA
PTPRE-R	GTCGACAGGAATGAAATTAAGAGATTTAAAG
RMI1-F	CTCGAGTTTGCTGATTATAATATTTGGTATA
RMI1-R	GTCGACGACACCTAAAGACATAGATAAATGC
SEMA3A-F	CTCGAGAGCAAAGCCATTGTATTGAACTAAA
SEMA3A-R	GTCGACATGTTTTTTTTTTTTTTTAATATTTCCA
SNX18-F	CTCGAGGGACGTTGGATTATGGACTTTTTCA
SNX18-R	GTCGACTTACAATGTGTGGTACTGTTTCTATTATAT
SYS1-F	CTCGAGCCTCTTTGAGGGTAATAACAGAATT
SYS1-R	GTCGACCACTGCAAAGGTAACTGCTGTGA
YTHDF3-F	CTCGAGTCGTCAAGCACTTTCTGTCCTGAA
YTHDF3-R	GTCGACAACTACAACAAAGAAAAAGTTGAAGC
SEMA3A-Mut-R	TAGGAACAATCCAACCACGTTTTG
SEMA3A-Mut-F	GTTCCTAATATTTTGTCTACCGGCC
