# Supplementary material for: Aberrant Expression of miR-362 Promotes Lung Cancer Metastasis through Downregulation of Sema3A
Source: J Immunol Res. 2018 Aug 1;2018:1687097. doi: 10.1155/2018/1687097 (PMC6093061; doi:10.1155/2018/1687097)
Supplement: Supplementary Materials — Supplementary Figure 1: miR-362 affects other functions in NSCLC. (A, E) Cell colony formation ability decreased when miR-362 was absent in A549. (C, G) Cell colony formation ability decreased when miR-362 was absent in 95-D. (B, F) Colony formation assays were employed in A549 cell line transfected by miR-362. (D, H) Colony formation assays were employed in 95-D cell line transfected by miR-362. miR-362 concentration was 50 nm. (I, J) The cell apoptosis rate changes when miR-362 was absent in A549 and 95-D. (K, L) The cell cycle changed when miR-362 was absent in A549 and 95-D. Data are from three independent experiments. Supplementary Figure 2: prediction of miR-362 target genes. (A) The prediction of miR-362 targets by miRanda, TargetScan, miRWalk, and miRDB software. (B) Reporter vector constructs containing the Sema3A portion of the 3′UTR with the miR-362 binding site and mutated binding site. Supplemental Table 1: qRT-PCR primers of genes. [file 1687097.f1.zip › Supplementary Figure 2_JIR_2356957.pptx]

## Slide 1
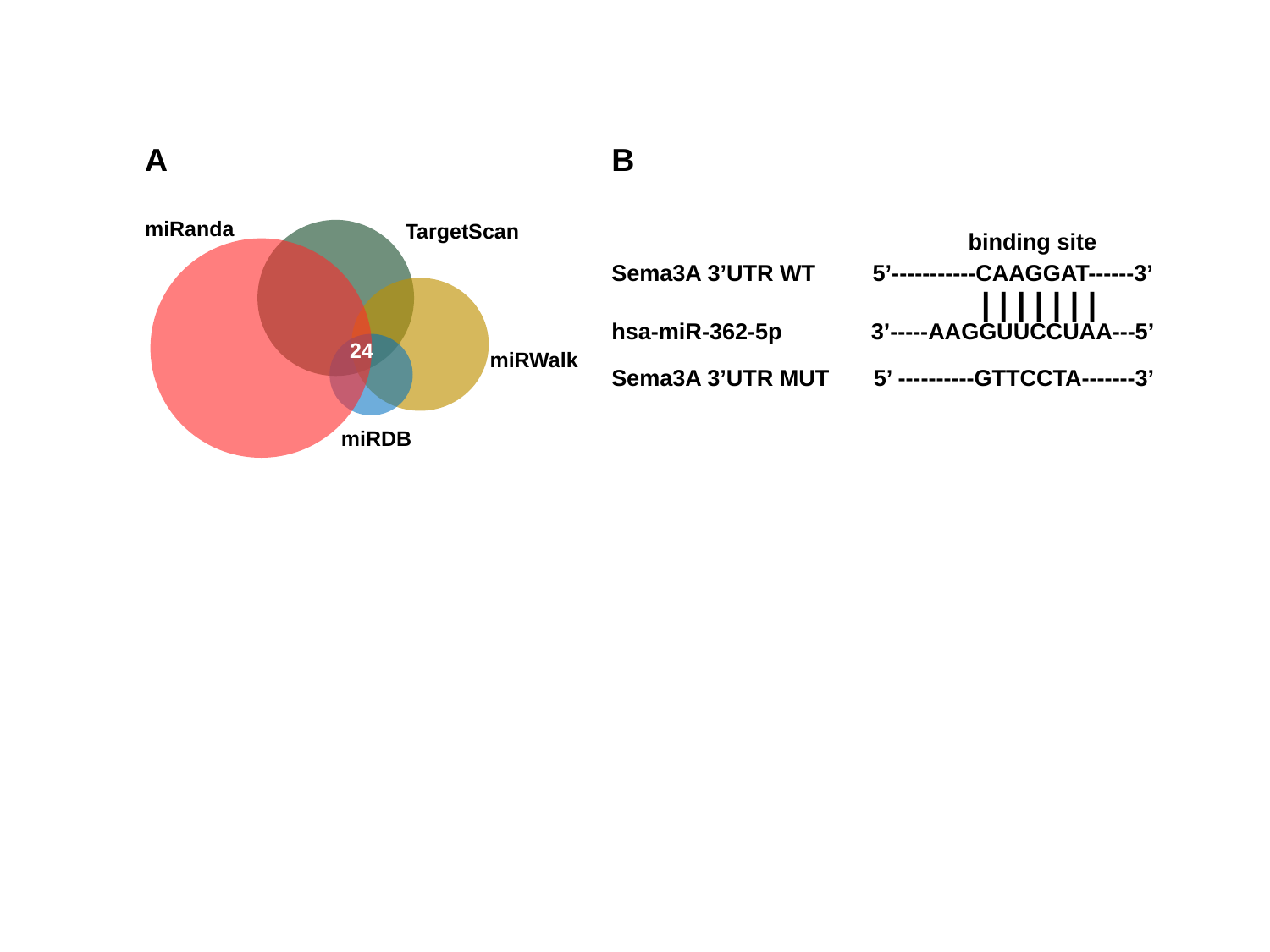

A
B
miRanda
TargetScan
24
binding site
Sema3A 3’UTR WT 5’-----------CAAGGAT------3’
| | | | | | |
hsa-miR-362-5p 3’-----AAGGUUCCUAA---5’
Sema3A 3’UTR MUT 5’ ----------GTTCCTA-------3’
miRWalk
miRDB
